# Comparative analysis of gene family evolution demonstrates expansion of digestive, immunity and olfactory functions in the black soldier fly (*Hermetia illucens*) lineage

**DOI:** 10.1038/s41437-025-00805-6

**Published:** 2025-10-23

**Authors:** Wenjun Zhou, Daniele Kunz, Chris D. Jiggins

**Affiliations:** 1 Department of Zoology, University of Cambridge, Cambridge, UK; 2 Department of Biochemistry, University of Cambridge, Cambridge, UK

**Keywords:** Comparative genomics, Genome evolution

## Abstract

Structural variants such as chromosomal rearrangements and gene duplications can play an important role in the adaptation and diversification of organisms. Here, we used comparative genomics to study the functional implications of structural variants across two families of flies. We compared the reference genomes of eight Asilidae species and six Stratiomyidae species, including the black soldier fly (*Hermetia illucens*), a species with an ability to convert organic waste into biomass and a recently expanded global range. Our results show that the genomes of Stratiomyidae are generally larger than Asilidae and contain a higher proportion of transposable elements, many of which are recently expanded. Gene families showing more gene duplications are enriched for life history related functions such as metabolism in Stratiomyidae which are known to be active decomposers, and longevity in Asilidae which are predators and have generally longer lifespan than Stratiomyidae. Gene families showing more gene duplications that are specific to *H. illucens* are mostly related to olfactory and immune responses, while across the Stratiomyidae there is enrichment of digestive and metabolic functions such as proteolysis, providing an explanation for the higher decomposing efficiency and adaptive ability of *H. illucens* compared to other Stratiomyidae species in decomposing environments. Together, our results shed light on the contribution of structural variants to functional adaptation and gene family expansions that have likely played a role in the ecological success of the black soldier fly.

## Introduction

Structural variations (SVs) are large scale variations in sequence (typically >50 bp) caused by insertions, deletions, inversions, duplications and sequence changes due to transposable elements (TEs) (Alkan et al. 2011; Berdan et al. 2024). As a major component of variation in the genome, SVs can play an important role in adaptation and speciation. For example, several inversions between *Drosophila pseudoobscura* and *D. persimilis* are associated with hybrid sterility, maintaining reproductive isolation (Noor et al. 2001; Zhang et al. 2021). In the Colorado potato beetle *Leptinotarsa decemlineata*, SVs are related to insecticide resistance and facilitate the rapid adaptation of this pest species into new agricultural environments (Cohen et al. 2023). In Lepidoptera, SVs play a role in adaptive wing pattern polymorphism (Joron et al. 2011; Brien et al. 2023) and the dynamics of gene family expansion and contraction are associated with diet breadth in butterflies (Dort et al. 2024).

Among many types of SVs, gene copy number variation (CNV) which includes the gain and loss of gene copies, is an important source of genetic variation that can contribute to adaptation. Duplication and deletion of preexisting genes can generate considerable genetic variation and is an important source of variation in genomes (Katju and Bergthorsson 2013). Gene duplications can lead to multiple outcomes. On one hand, duplications of a single gene can create functional redundancy and may affect gene dosage (Hahn 2009; Magadum et al. 2013; Kuzmin et al. 2022). Duplicated genes can also either become a pseudogene, or be subfunctionalized, whereby both genes adopt parts of their original function (Magadum et al. 2013). Alternatively, redundancy can also help the newly duplicated genes “escape” from purifying selection and evolve new functions, known as neofunctionalization (Hahn 2009; Magadum et al. 2013; Birchler and Yang 2022; Kuzmin et al. 2022).

Here we focus on SVs and their functional roles in the black soldier fly (*Hermetia illucens*) lineage, a Dipteran species of commercial interest due to its ability to convert organic waste into biomass. *Hermetia illucens* belongs to the Stratiomyidae family (soldier flies), which consists of over 2700 species found around the world (Woodley and Thompson 2001). Stratiomyidae larvae are often found in water or damp substrates such as decaying organic matter. Despite the similar ecology of many Stratiomyidae species, *Hermetia illucens* stands out as the only species that has spread around the globe as a human commensal and is associated with industrial uses in waste treatment (Nguyen et al. 2015; Tomberlin and van Huis 2020; Siddiqui et al. 2022).

In this study, we used chromosome-level reference genomes of six Stratiomyidae species and eight Asilidae species to explore the potential correlation between genome structure and their life history differences. During the larval stage, Asilidae are often found in soil or other damp substrates such as rotting organic matter similar to those of Stratiomyidae larvae. However, unlike the Stratiomyidae, Asilidae species are long-lived, with a life span ranging from one to three years, while the Stratiomyidae usually only have short life cycles. Compared to Stratiomyidae adults which usually only feed on plant liquids or do not feed at all, Asilidae adults are predators and feed on other insects. On the phylogeny, Asilidae are one of the families in superfamily Asiloidea, which is sister clade to the superfamily Stratiomyomorpha that contains Stratiomyidae (Wiegmann et al. 2011), making this an interesting phylogenetic and phenotypic comparison.

We address the following questions: (1) How much variation in genome size and gene number is there between Stratiomyidae and Asilidae? (2) What is the pattern of types and proportion of repetitive elements among Stratiomyidae and Asilidae species? (3) What are the dynamics of gene birth and death across the phylogeny? (4) How is gene family expansion related to species-specific life history and functional variation?

## Materials and methods

### Obtaining and pre-processing reference genomes

All genome assemblies were downloaded from the NCBI database with their RefSeq assembly numbers before 1st March 2025. Annotations in GFF format and peptide sequences of the reference genomes (McCulloch et al. 2023; Thomas et al. 2023; Crowley and Garland et al. 2024; Crowley and Sivell et al. 2024; Crowley and Akinmusola et al. 2024a; Crowley, University of Oxford and Wytham Woods Genome Acquisition Lab et al. 2024; Crowley and Akinmusola et al. 2024b; Mitchell et al. 2024a; Mitchell et al. 2024b; Nash et al. 2024; Sivell, Sivell, Natural History Museum Genome Acquisition Lab, et al. 2024; Sivell and McAlister et al. 2024; Sivell, Sivell, Mitchell, et al. 2024) were obtained from the Darwin Tree of Life Project (https://wellcomeopenresearch.org/treeoflife) through its online data portal (https://www.darwintreeoflife.org/genomes/genome-notes/) except for *Hermetia illucens* and *Drosophila melanogaster* whose GFF annotations and protein sequences were downloaded from their NCBI RefSeq FTP archive.

### Genome quality assessment

To evaluate the completeness of reference genomes assembled via different pipelines, we used BUSCO 5.8.2 (Simão et al. 2015) to summarize genome completeness and assembly quality before actual analysis. All genomes were compared to the Diptera database diptera_odb10 downloaded from BUSCO website (https://busco-data.ezlab.org/v4/data/lineages/diptera_odb10.2019-11-20.tar.gz). Summary plot was generated using script generate_plot.py implemented in BUSCO based on the output text file of each genome.

All annotation files were filtered with the implemented python script in OrthoFinder software (https://github.com/davidemms/OrthoFinder/blob/master/tools/primary_transcript.py) to keep only the longest transcript of each gene in each genome. All annotations downloaded were checked for the consistency of gene names to remain an unique naming format for each genome for easy identification in downstream analyses.

### Repetitive elements identification

We used Earl Grey 5.1.1 (Baril et al. 2024), a pipeline in which RepeatMasker (Tarailo-Graovac and Chen 2009) and RepeatModeler2 (Flynn et al. 2020) is automatically called, to identify repetitive elements on each reference genome. All genomes were used for de novo TE identification using RepeatModeler2 implemented in the pipeline. For each genome, Earl Grey was run with ten iterations of its “BLAST, Extract, Align, Trim” process (https://github.com/jamesdgalbraith/TEstrainer). The de novo TE library of each genome was used for TE annotation using RepeatMasker. LTR_Finder (Xu and Wang 2007; Ou and Jiang 2019) was also used and the output was combined with previous RepeatMasker annotation using RepeatCraft (Wong and Simakov 2019). Final output and TE annotations of each genome were used for Kimura distance calculation using script “divergence_calc.py” implemented in Earl Grey. A summary bar chart was generated using ggplot2 R package (Wickham 2016).

### Orthogroups identification, phylogeny construction and genome-wide synteny analysis

To evaluate orthology relationships among coding genes, we used OrthoFinder 2.5.5 (Emms and Kelly 2019) to assign protein coding genes in all selected 14 species into orthogroups. 201,275 genes (95.3% of total) were assigned to 15,964 orthogroups by OrthoFinder. Fifty percent of all genes were in orthogroups with 15 or more genes (G50 was 15) and were contained in the largest 4780 orthogroups (O50 was 4780). There were 6653 orthogroups with all species present and 3328 of these consisted entirely of single-copy genes.

When running OrthoFinder, maximum likelihood trees were inferred using multiple sequence alignments method (argument “-M msa”). Under this setting, the species tree is inferred using a concatenated multiple sequence alignment (MSA) of single-copy genes, which is based on the output of the OrthoFinder run. A species tree was constructed using the STAG method (Emms and Kelly 2018) with default setting. A total of 3328 orthogroups with single-copy genes in all species were used for the phylogeny construction. Species tree was visualized using FigTree 1.4.4 (http://tree.bio.ed.ac.uk/software/figtree/).

The results from OrthoFinder were then used as the input for GENESPACE 1.2.3 (Lovell et al. 2022) to construct synteny plots. Two steps of format conversion were performed before running GENESPACE. First, all GFF annotation files were reformatted into bed files using “convert2bed” function (version: 2.4.39) in BEDOPS (Neph et al. 2012). Only gene name, start and end position were kept for each gene in the final bed files. Second, the peptide sequences in fasta format were renamed using corresponding gene names in the bed file for each species. The raw output file folder of OrthoFinder was assigned to GENESPACE using the “rawOrthofinderDir” parameter in GENESPACE R command. The genome of *Hermetia illucens* was used as reference in the plotting pipeline, and chromosomes of all other genomes were arranged based on the order of *H. illucens* chromosomes (“1”, “2”, “3”, “4”, “5”, “6”, “X”).

### Gene family expansion and contraction analysis

A CAFE5 (Mendes et al. 2021) pipeline was used to calculate the gene family evolutionary rate Lambda (λ). The species tree constructed by OrthoFinder was first converted into an ultrametric tree using r8s (Sanderson 2003). A calibration of divergence time of 165 million years ago (MYA) was set between *Drosophila melanogaster* and *Hermetia illucens* based on a previous study (Wiegmann et al. 2011). Input control file for r8s was generated using the implemented python script in CAFE5 (https://github.com/hahnlab/CAFE5/blob/master/docs/tutorial/prep_r8s.py). The output tree file of r8s was used as the input of CAFE5 pipeline to estimate the evolution rate of gene families.

We used two types of models in CAFE5 to find the best fit of the dataset. First, three base models were used to determine the appropriate number of Lambda values: a model where all terminal and non-terminal nodes across the phylogeny share a single Lambda value (number of Lambda: 1), a model where Stratiomyidae, Asilidae and outgroup share their own Lambda value (number of Lambda: 3), and a model in which each node has an unique Lambda value (number of Lambda: 28). Each model setting was run three times, and the second model setting was chosen for the next round of testing based on its highest final likelihood (Supplementary Table 1). In the second round of test run, Gamma models instead of base models were used to allow different gene families to belong to different evolutionary rate categories, where the Gamma value represents the number of categories. The Gamma value (-k) was set as 1, 2, 3, 4 and 5 for each model, and for all models, Stratiomyidae, Asilidae and outgroup share their own Lambda value (number of Lambda: 3). Each model was run three times, and the one with 5 Gamma categories had the highest average likelihood (Supplementary Table 1). The best model setting was then run for another seven times. Five out of the ten runs had high overall failure rate where any gene family has failure rates >20%, and thus were not considered when choosing the best run. The run with the highest likelihood and without high failure rate was chosen as the final result (Replicate 10, Supplementary Table 1). All raw output files in the output directory of CAFE5 were used as input of CafePlotter (https://github.com/moshi4/CafePlotter) for visualization.

### Functional enrichment analysis

Duplicated genes were extracted from the OrthoFinder output and used for functional enrichment analysis in the Database for Annotation, Visualization, and Integrated Discovery (DAVID) (Sherman et al. 2022). For *Hermetia illucens* genes, gene IDs in its annotation were used directly as the input gene list. For orthogroups with duplications on the most recent common ancestor nodes of Stratiomyidae and Asilidae, enrichment analyses were performed using their orthologue gene IDs in the *Drosophila melanogaster* annotation.

Outputs from the enrichment analysis were downloaded as text files and visualized with jvenn (Bardou et al. 2014) and ggplot2.

## Results

### Genome size expansion in Stratiomyidae

We first summarized BUSCO scores of all reference genomes to explore consistency between genomes assembled and annotated by different pipelines. After being compared to a total database of 3285 Dipteran BUSCO genes, the dataset of 14 reference genomes reached an average of 96.20% completeness (SD = 0.0102), showing consistent level of assembling quality across multiple pipelines (Supplementary Fig. 1).

On average, Stratiomyidae (mean genome size = 0.721 gigabytes) have larger genomes compared to Asilidae (mean genome size = 0.559 gigabytes) (Table 1, Supplementary Fig. 2). However, *Dioctria linearis* and *D. rufipes* from the Asilidae family have the largest genomes of all species in the study. Average gene number of Stratiomyidae (15,972.83) species also exceeds the one of Asilidae (11,714.25) with the exceptions of *Stratiomys singularior* and *Beris chalybata*. Interestingly, despite having a large genome, the two *Dioctria* species do not possess more genes than other Asilidae species in the dataset (Table 1).

**Table 1:** Genome information, BUSCO scores, sequencing technology and annotation method of the species used in this study.

Family	Species	NCBI assembly number	Size/Gb	Chromosome number	Scaffold number	Gene number	Complete BUSCO gene number	Fragmented BUSCO gene number	Missing BUSCO gene number	Complete BUSCO percentage	Sequencing technology	Annotation method
Asilidae	*Leptogaster cylindrica*	GCA_963082835.1	0.185	5	187	10,816	3171	10	104	0.955	PacBio, Hi-C	Ensembl Genebuild annotation system (Aken et al. 2016) with RNA-Seq and UniProt protein data
	*Neoitamus cyanurus*	GCA_947538895.1	0.345	10	16	12,046	3184	33	68	0.962	PacBio, Hi-C	Ensembl Genebuild annotation system (Aken et al. 2016) with RNA-Seq and UniProt protein data
	*Eutolmus rufibarbis*	GCA_963920795.1	0.27	7	8	12,655	3191	34	60	0.962	PacBio, Hi-C	Ensembl Genebuild annotation system (Aken et al. 2016) with RNA-Seq and UniProt protein data
	*Machimus rusticus*	GCA_951509405.1	0.264	8	8	11,510	3195	31	59	0.967	PacBio, Hi-C	Ensembl Genebuild annotation system (Aken et al. 2016) with RNA-Seq and UniProt protein data
	*Tolmerus cingulatus*	GCA_959613345.1	0.264	6	148	12,047	3204	27	54	0.969	PacBio, Hi-C	Ensembl Genebuild annotation system (Aken et al. 2016) with RNA-Seq and UniProt protein data
	*Machimus atricapillus*	GCA_933228815.1	0.253	6	28	10,978	3194	30	61	0.958	PacBio, Hi-C	Ensembl Genebuild annotation system (Aken et al. 2016) with RNA-Seq and UniProt protein data
	*Dioctria linearis*	GCA_963930735.1	1.521	5	3127	12,074	3170	45	70	0.955	PacBio, Hi-C	Ensembl Genebuild annotation system (Aken et al. 2016) with RNA-Seq and UniProt protein data
	*Dioctria rufipes*	GCA_963924295.1	1.369	6	1456	12,218	3179	45	61	0.956	PacBio, Hi-C	Ensembl Genebuild annotation system (Aken et al. 2016) with RNA-Seq and UniProt protein data
Stratiomyidae	*Stratiomys singularior*	GCA_954870665.1	0.674	6	404	11,614	3107	49	129	0.939	PacBio, Hi-C	Ensembl Genebuild annotation system (Aken et al. 2016) with RNA-Seq and UniProt protein data
	*Microchrysa polita*	GCA_949715475.1	0.798	8	98	19,601	3105	69	111	0.938	PacBio, Hi-C	BRAKER2 with no RNA-Seq data
	*Hermetia illucens*	GCF_905115235.1	0.948	7	20	14,004	3116	67	102	0.942	PacBio, Hi-C	BRAKER2 with RNA-Seq data
	*Chorisops tibialis*	GCA_963669355.1	0.816	5	15	22,148	3147	70	68	0.947	PacBio, Hi-C	BRAKER2 with no RNA-Seq data
	*Beris morrisii*	GCA_951812415.1	0.578	5	33	18,031	3157	56	72	0.954	PacBio, Hi-C	BRAKER2 with no RNA-Seq data
	*Beris chalybata*	GCA_949128065.1	0.511	6	11	10,439	3124	62	99	0.942	PacBio, Hi-C	BRAKER2 with no RNA-Seq data

**Fig. 1:**
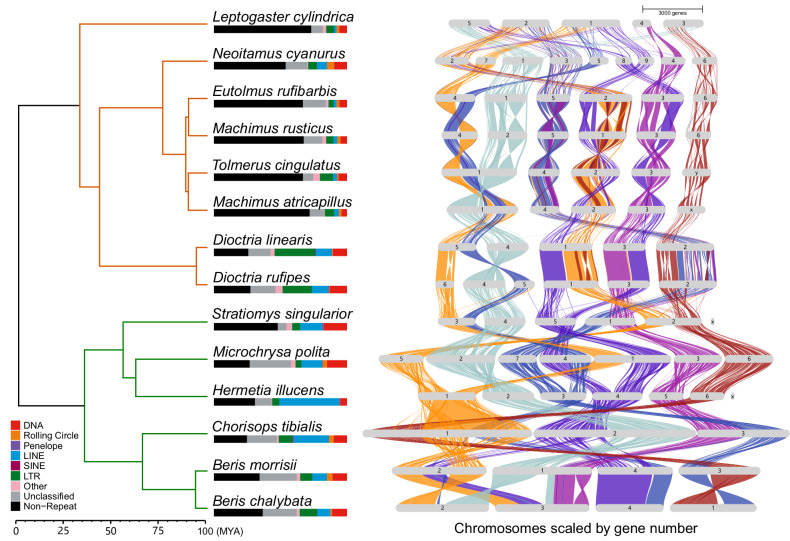
Species tree, proportion of transposable elements (TEs) and genome-wide synteny scaled by gene number on the genomes. Syntenic blocks are divided and aligned based on the order of chromosomes in *Hermetia illucens*. The species tree was rooted using outgroup *Drosophila melanogaster*, which is not shown in the tree. Barplots at the bottom of species names represent the proportion of different types of transposable elements on the genome. Simple repeats, microsatellites and repeat RNAs were combined as “Other” in the barplots. Stratiomyidae and Asilidae families were marked with green and orange branch colors in the phylogeny, respectively. The species tree was calibrated based on estimated divergence time between *Drosophila melanogaster* and Stratiomyidae (MYA) (Wiegmann et al. 2011).

Genome synteny analysis shows many chromosomal rearrangements across the phylogeny (Fig. 1, Supplementary Fig. 2). Even between species from the same genus there are a large number of inversion, fission and fusion events (Supplementary Table 2).

### Variation of repetitive elements across the phylogeny

In general, Stratiomyidae have a higher proportion of repetitive elements compared to most Asilidae species except for the two *Dioctria spp*. (Fig. 1, Supplementary Table 3). The proportion of repetitive elements in the genome shows a moderate negative correlation with BUSCO completeness (Pearson’s *r* = −0.63, *p* = 0.015) (Supplementary Fig. 3A), indicating potential effects of assembly quality on the repeat identification process. However, compared to BUSCO completeness, the correlation between genome size and TE proportion is stronger (Pearson’s *r* = 0.84, *p* = 0.00018) (Supplementary Fig. 3B). There is considerable variation in types of repetitive elements across the phylogeny. For example, long terminal repeats (LTRs) are common in Asilidae genomes, but not in Stratiomyidae. Among Stratiomyidae, DNA repeats dominated in *Stratiomys singularior* and *Microchrysa polita*, but were a relatively low proportion of the repeats in *Hermetia illucens*, where long interspersed nuclear elements (LINEs) were more common compared to other subclasses (Fig. 1, Supplementary Table 3).

When divided into subclasses, the majority of repetitive elements show a pattern of recent activity, indicated by the peaks of closely related copies near right side on the X-axis (Supplementary Fig. 4). For all species in the dataset, the dominant repetitive elements tend to show lower Kimura 2-Parameter distances (Supplementary Fig. 4), showing recent emergence of those repeats. Although the proportion of TEs are generally larger in Stratiomyidae than in Asilidae (Welch Two Sample t-test, *p* = 0.01317), the types and divergence time of TEs do not show any strong phylogenetic pattern consistent with very rapid turnover.

### Gene duplication events and gene family evolution

Using *Drosophila melanogaster* as an outgroup, 201,275 out of 211,239 genes (95.3%) were assigned into 15,964 orthogroups, among which 1707 were species-specific with orthologues found in only one species, and 6653 had orthologues in all selected species in the dataset (Supplementary Table 4).

**Fig. 2:**
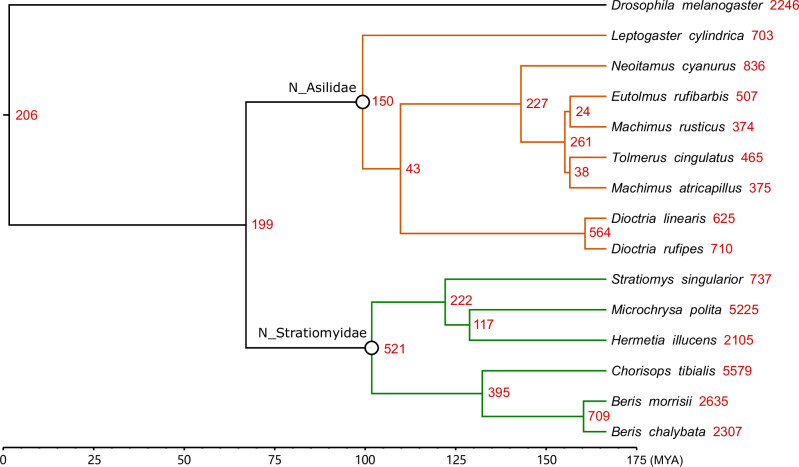
Gene duplication events across the phylogeny. Numbers of duplications on each node are marked on the right side of the node. Only gene duplication events with support higher than 0.5 were considered. The node of the most recent common ancestor of Stratiomyidae and Asilidae were marked as “N_ Stratiomyidae” and “N_Asilidae”. Stratiomyidae and Asilidae families were marked with green and orange branch colors in the phylogeny, respectively. The species tree was calibrated based on estimated divergence time between *Drosophila melanogaster* and Stratiomyidae (MYA) (Wiegmann et al. 2011).

Gene duplication events were counted for each of the orthogroups. 32,493 gene duplication events were identified across the phylogenetic tree (Fig. 2, Supplementary Table 5). Similar to the TE proportion, gene duplication events also showed a moderate negative correlation with BUSCO completeness (Pearson’s *r* = −0.61, *p* = 0.019) (Supplementary Fig. 3C), while the number of gene duplication events identified is strongly correlated with the total number of genes (Pearson’s *r* = 0.90, *p* = 0.0000093) (Supplementary Fig. 3D).

Most of the duplications were on terminal nodes. Compared to Asilidae, Stratiomyidae have more gene duplication events, particularly on terminal nodes. *Stratiomys singularior* has the lowest number of terminal duplication events among the Stratiomyidae species.

**Fig. 3:**
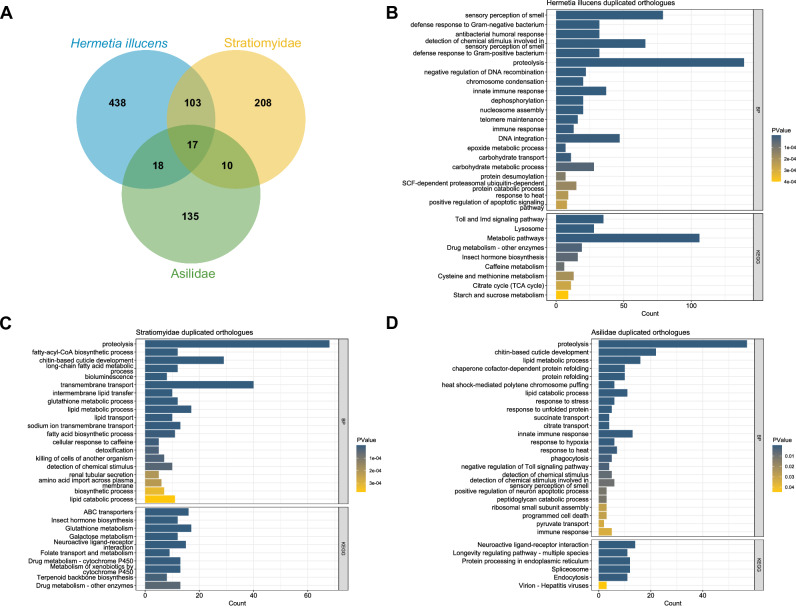
Overlap and functional annotation of duplicated orthogroups among *Hermetia illucens*, Stratiomyidae and Asilidae. **A** Venn diagram of orthogroups with duplication events. **B** GO biological process and KEGG enriched functional terms of *Hermetia illucens* specific duplicated genes. **C** GO biological process and KEGG enriched functional terms of duplicated genes in the most recent common ancestor node of Stratiomyidae. **D** GO biological process and KEGG enriched functional terms of duplicated genes in the most recent common ancestor node of Asilidae. The length of the bars represents number of genes that are enriched onto each functional term, and *p* value was represented by color.

To study the function of duplicated genes, genes within orthogroups with duplication events showing support values > 0.5 on the most recent common ancestor node of Stratiomyidae (“N_ Stratiomyidae” in Fig. 2) and Asilidae (“N_ Asilidae” in Fig. 2) were extracted and GO and KEGG enrichment analyses were performed on those genes. Although several functional terms showing gene duplications were shared between families, the numbers of overlapping orthogroups was limited. Only 27 orthogroups were shared among the 180 orthogroups that had high support for duplication events in the common ancestor of Asilidae and 338 in Stratiomyidae (Fig. 3A). There was greater overlap between orthogroups with duplications in the common ancestor of Stratiomyidae and those in *Hermetia illucens* (Fig. 3A). After excluding those orthogroups that are shared, duplicated genes on terminal node of *Hermetia illucens* showed a different pattern compared to those duplicated in the common ancestor of Stratiomyidae. Aside from proteolysis which still had the highest number of enriched genes, more genes were enriched in immune and antibacterial responses and olfaction (Fig. 3B). In KEGG pathways, the most enriched terms are metabolism-related, while “Toll and Imd signaling pathway”, involved in the immune response, is also enriched (Fig. 3B). These terms are consistent with adaptation to the decomposing-related life history of *H. illucens*.

In both Stratiomyidae and Asilidae, the enriched biological process term with the highest gene count is “proteolysis” (GO:0006508, *p* = 3.43E-19 for Stratiomyidae and *p* = 1.66E-29 for Asilidae). “Lipid metabolic process” (GO:0006629, *p* = 1.01E-06 for Stratiomyidae and *p* = 1.55E-10 for Asilidae) and “lipid catabolic process” (GO:0016042, *p* = 3.73E-04 for Stratiomyidae and *p* = 4.18E-07 for Asilidae) are also present in both families. Duplicated genes in the common ancestor of Stratiomyidae are mainly enriched for metabolism related biological processes and pathways (Fig. 3C, Supplementary Table 6), while duplicated genes in the common ancestor of Asilidae are more enriched for functions related to responses to environmental stimulations, longevity regulation and protein refolding (Fig. 3D, Supplementary Table 6).

**Fig. 4:**
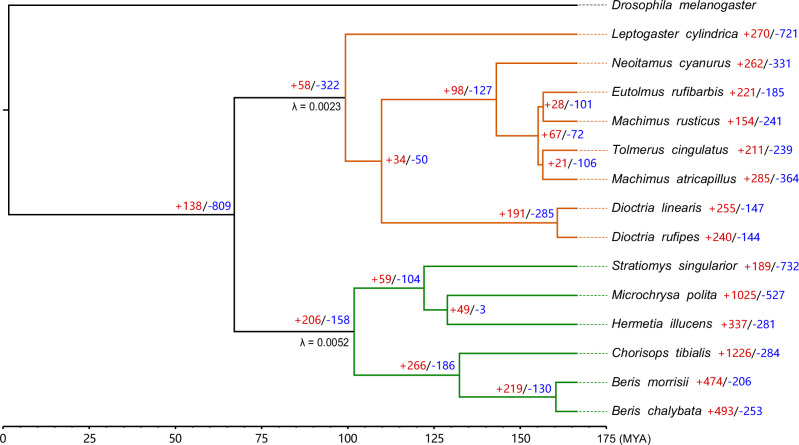
Gene family evolution across the phylogeny. Number on the left side of each node represents the gene birth-death parameter λ of this node. Numbers with “+” and “-” beside each node represent the total number of expanded (“+”) and contracted (“−”) orthogroups on this node. Stratiomyidae and Asilidae families were marked with green and orange branch colors in the phylogeny, respectively. The species tree was calibrated based on an estimated divergence time between *Drosophila melanogaster* and Stratiomyidae (MYA) (Wiegmann et al. 2011). Dashed lines are not included as a part of the branch length.

We used the gene family evolutionary rate Lambda (λ), which is the probability of any gene to be gained or lost in a gene family, as calculated in the CAFE5 pipeline (Mendes et al. 2021), to infer the pattern of expansion and contraction of orthogroups across the phylogeny. Each orthogroup assigned in the previous analysis is considered as a “gene family” and its size was mapped to each node on the species tree. Two types of models were tested in the CAFE5 pipeline: the default base model and the Gamma model where each gene family is allowed to belong to a different evolutionary rate category. After test runs with three independent replicates, the model that best fit the dataset is a 3-category Gamma model where Stratiomyidae, Asilidae and outgroup (*Drosophila melanogaster*) have their own Lambda values, and each node within the same Stratiomyidae or Asilidae shares the same Lambda (Supplementary Table 1). Under this model, the Lambda value for Stratiomyidae is 0.0052, and the Lambda value for Asilidae is 0.0023 (Fig. 4), suggesting a higher gene family evolutionary rate in Stratiomyidae species.

In *Hermetia illucens*, 70 orthogroups showed significant (*p* < 0.05) changes from the last common ancestor node (Supplementary Table 7), most of which are expanded but not contracted. These significantly expanded orthogroups include several gene families involved in immune response (OG0000160 and OG0000220) and olfaction (OG0000020 and OG0000025) (Supplementary Table 7, Supplementary Table 8).

In the top twenty expanded orthogroups in *Hermetia illucens*, only one (OG0000005) has a lower copy number compared to its closest relative *Microchrysa polita* (Supplementary Table 8). In general, when compared to the most recent common ancestor node of Stratiomyidae, many of these orthogroups expanded in both species, but their expansions in *H. illucens* are larger than in *Microchrysa polita*.

## Discussion

The black soldier fly *Hermetia illucens* has become a major commercial insect species used to consume organic waste. In addition to its value in the industry, it also provides an interesting example of rapid range expansion and human-mediated evolution. By comparing high quality chromosome-level reference genomes from the Stratiomyidae and Asilidae families, we provide evidence for the adaptation of *H. illucens* and its close relatives to their specific life history. We have shown recent activity of TEs, as well as large amounts of gene duplication on terminal nodes, high gene birth-death rate and expansions of metabolism and immune related gene families.

The quality of genome assembly can impact downstream analysis in comparative genomic studies (Florea et al. 2011; Mariene and Wasmuth 2025). In our study, most genomes used in analyses were generated using the same pipeline in Darwin Tree of Life project (https://www.darwintreeoflife.org/) except *Hermetia illucens* and *Drosophila melanogaster* (Table 1). However, the annotations of *Microchrysa polita*, *Chorisops tibialis*, *Beris morrisii* and *Beris chalybata* were generated with no RNA-Seq data (Table 1). There was also slight variation in the BUSCO completeness in the dataset (Table 1). This variation shows moderate negative correlation with both TE proportion and gene duplication events (Supplementary Fig. 3A, C), indicating potential effects of assembly quality on SV detection, such as fragmented gene sections being identified as inserted repetitive elements or duplicated orthologues. However, genome size shows higher Pearson correlation coefficient in the linear regression analysis with TE proportion compared to BUSCO completeness alone (Supplementary Fig. 3A, B). A similar pattern was shown in the correlation of BUSCO completeness and total gene number with gene duplication events (Supplementary Fig. 3C, D). Although some effects of assembly quality are likely, their extent remains difficult to disentangle from other factors in the current dataset.

TEs have been suggested to be an important factor driving insect evolution (Gilbert et al. 2021). Zhou et al. found that genome size expansion is associated with its TE proportion, and the expansion of TEs are enabled by DNA methylation (Zhou et al. 2020). The accumulation of TEs might also appear during geographical range expansion (Jiang et al. 2024). We found varying proportions of TEs ranging from 26.85% of the genome in *Leptogaster cylindrica* to 75.18% in *Chorisops tibialis* (Supplementary Table 3), which corresponds to previous findings in Diptera where proportion of TEs largely varies across the phylogeny (Petersen et al. 2019). The TEs in Stratiomyidae and Asilidae not only vary in abundance but also in divergence time. In all species, DNA, LINEs and LTRs show recent activity which can be represented by the peaks of base pair numbers with Kimura 2-Parameter Distance near zero (near right side on the X-axis, Supplementary Fig. 4). This suggests a recent expansion of these TEs in both Stratiomyidae and Asilidae families. In *Hermetia illucens*, the activity of LINEs is consistent across the X-axis (Supplementary Fig. 4K), suggesting that this TE superfamily is not only present on a recent timescale, but has always been a major component of the TEs in the *H. illucens* genome through its evolutionary history. However, in *Chorisops tibialis* (Supplementary Fig. 4L), SINEs also show activity with higher Kimura 2-Parameter Distance, while the same TE superfamily does not exist in *Dioctria rufipes* (Supplementary Fig. 4H), *Stratiomys singularior* (Supplementary Fig. 4I), and *Beris morrisii* (Supplementary Fig. 4M). In general, the types of TEs in Stratiomyidae and Asilidae correspond to phylogenetic relationships, with more similar proportion of TE types are found between more closely related species (Fig. 1), which is consistent with previous findings in the majority of insects (Gilbert et al. 2021). However, the distribution of Kimura 2-Parameter Distance of different TEs types does not show a similar phylogenetic pattern, consistent with the patterns found in *Drosophila* (Petersen et al. 2019). Despite the limitation of sample size and coverage of the taxa in our study, there was a significant positive linear correlation between TE proportion and genome size in Stratiomyidae and Asilidae species (Supplementary Fig. 3B), indicating a likely contribution of TE expansion to genome size changes in the two families, consistent with previous findings in other organisms (Marburger et al. 2018; Naville et al. 2019; Wong et al. 2019; Zhou et al. 2020; Oggenfuss et al. 2021). The recent expansion of TEs may also contribute to adaptation to new environments and stress responses (Chénais et al. 2012), which are also indicated by the enriched functional terms in Fig. 3.

Domestication represents an excellent system in which to study recent adaptation. *Hermetia illucens* is a putative example of recent domestication, with populations brought into captivity repeatedly around the world. Changes of food supply often lead to metabolism-related functional shifts in domesticated animals (Gering et al. 2019). In other species, domestication is associated with changes in diet (Luca et al. 2010; Axelsson et al. 2013), immunity (Chen et al. 2017) and digestive system gene functions (Axelsson et al. 2013; Chen et al. 2017; Pajic et al. 2019; Glazko et al. 2021). The individual of Her*metia illucens* that was sequenced for the reference genome was from an industrial strain (Generalovic et al. 2021), so we cannot here distinguish between adaptations associated with the species generally and those that have happened since its domestication. It is possible that duplicated genes related to carbohydrate metabolic process that are not shared by other Stratiomyidae species (Fig. 3B) are related to the recent domestication of *H. illucens*, involving adaptation to a more starch-rich diet, similar to previous findings in other domesticated mammals (Axelsson et al. 2013; Li et al. 2013; Ollivier et al. 2016; Reiter et al. 2016; Lye and Purugganan 2019; Pajic et al. 2019). In addition to carbohydrate, several other metabolic pathways can also be found in the enriched functional terms, such as drug, cysteine, methionine, sucrose and even caffeine metabolism (Fig. 3B) in *H. illucens*. These enriched pathways could be associated with higher efficiency of *H. illucens* on certain diets compared to other Stratiomyidae species, and potential influences of the domestication process. Two major functions, olfactory sensory and immune responses, are specifically enriched in *H. illucens* compared to the most recent common ancestor node of Stratiomyidae (Fig. 3A, Table 2). Two families of olfactory genes, odorant receptor 2a, 33b, and 59a (*Or2a*, *Or33b* and *Or59a*, OG0000020) and general odorant-binding protein 99a (*Obp99a*, OG0000025), both show double the copy number in *H. illucens* compared to its close relative *Microchrysa polita* (Supplementary Table 8). Similar patterns can also be found in the peptidoglycan recognition protein 3 (*PGLYRP3*, OG0000136), cecropin (*CecA1*, OG0000160) and gram-negative bacteria-binding protein 3 (*GNBP3*, OG0000220) (Supplementary Table 8). Similar pattern can also be found in the coding genes of peptidoglycan recognition protein 3 (*PGLYRP3*, OG0000136), cecropin (*CecA1*, OG0000160), and gram-negative bacteria-binding protein 3 (*GNBP3*, OG0000220) (Supplementary Table 8). The expansion of olfactory and immune related genes in *H. illucens* can potentially explain the reason that it has become so widely used in bioconversion of organic waste (Tomberlin and van Huis 2020) and is often seen as “dominant” species in compost piles. Besides, the enrichment of immune-related functions might also contribute to the symbiosis between *H. illucens* and its diverse range of gut microbiota (Jiang et al. 2019; Zhan et al. 2020; Eke et al. 2023; Luo et al. 2023; Yu et al. 2023). All these offer clues into how *H. illucens* has adapted to human influenced diets and a composting environment compared to its Stratiomyidae relatives.

Despite the differences in both adult diets, habitats and life span, the top hit in biological process GO terms is proteolysis (Fig. 3D) for both Stratiomyidae and Asilidae ancestral nodes. This suggests that some specific gene families and their function may have been involved in adaptation to different life history traits. The same pattern can be observed for lipid metabolic process (Fig. 3C, D). However, more enriched functional terms in Asilidae are related to responses to external stimuli, including response to stress, heat and hypoxia (Fig. 3D). One of the top hits in the KEGG pathway category in Asilidae, longevity regulating pathway (Fig. 3D), is not enriched for genes that are duplicated in Stratiomyidae, which may be associated with the longer life span of adults in Asilidae compared to Stratiomyidae.

Among the gene families that had significantly changed in *H. illucens*, the largest expansion appears in the *CYP* (cytochrome P450) gene family (Supplementary Table 7). As the only *CYP*-related one in the top 20 expanded orthogroup in the *H. illucens* lineage, OG0000004 experienced recurrent expansions from the common ancestor node between Stratiomyidae and Asilidae to the terminal node of *H. illucens* (Supplementary Fig. 5), with 66 copies in *H. illucens* compared to 35 in its close relative *Microchrysa polita*. The *CYP* family has been studied as a major contributor to insect genome evolution (Feyereisen 2006), involved in important functions such as ecdysteroid metabolism (Feyereisen 1999; Iga and Kataoka 2012), detoxification (Scott et al. 1998; Chandor-Proust et al. 2013; Cui et al. 2016; Lu et al. 2021; Xing et al. 2021) and sex pheromone biosynthesis (Fujii et al. 2020; Wang et al. 2024). Among the 66 expanded OG0000004 copies, 59 of them locate within a single region from 173348395 bp to 174874698 bp on Chromosome 1. Except for *LOC119646713* that was not characterized in the annotation from the reference genome, these genes all belong to the *CYP3* clade of *CYP* genes, which have been found to be most abundant among the *CYP* clades and have experienced recent expansions (Feyereisen 2006). Although the specific functions of *CYP* gene families in *H. illucens* are unknown, it is possible that the repeated expansions of these gene families during the speciation process played a role in its adaptation, especially to human-influenced diet and environment where resistance to toxins is essential to survival.

Apart from those gene families that were expanded, several gene families have been completely lost in *H. illucens* (Supplementary Table 7). One example is the gene family which includes *Or83c* and its orthologues (OG0000615). In *Drosophila melanogaster*, this odorant receptor is related to sensitivity to farnesol, a fruit rind volatile present in many ripe citrus rinds (Ronderos et al. 2014). The fact that this gene and its orthologues were lost while other groups of olfactory receptor genes such as *Or2a*, *Or59a*, *Or33a*, *Or33b*, *Or33c*, *Obp44a*, *Obp83g*, *Obp99a*, *Obp99b* and *Obp99c* are largely expanded in *H. illucens* suggests potential species-specific diet preference and diversification in olfactory related gene functions.

## Conclusions

Structural variants, and gene duplications in particular, are a driving force of functional adaptation. Using a comparative genomics approach, we show evidence of functional adaptation of the soldier flies (Stratiomyidae) and the robber flies (Asilidae) to their habitats and life history. We found a generally larger proportion of TEs in Stratiomyidae compared to Asilidae, and the majority of these are recently expanded. More gene duplication events were found on terminal nodes in Stratiomyidae than in Asilidae, and only 27 duplicated orthogroups are shared between the most recent common ancestors of these two families. We found 120 duplicated orthogroups that are shared between the most recent common ancestor of Stratiomyidae and *H. illucens*, indicating functional similarity of gene duplications. Certain genes involved in metabolism-related functions in *Hermetia illucens* are also duplicated in the common ancestor of Stratiomyidae, but more duplicated genes are related to olfactory sensory and immune responses in *H. illucens*, suggesting functional specialization in gene families that are beneficial to the decomposing life history in this lineage. Together, our results provide insights into the relationship between structural variation, especially gene duplications, and functional adaptation in two diverse families. We also provide directions for future experimental validation on these gene families and their specific role in functional pathways such as immune response, proteolysis and other metabolism.

## Data archiving

Not applicable. All raw data used in this study was obtained from public databases.

## Supplementary information

Supplementary Figure 1-5

Supplementary Table 1

Supplementary Table 2

Supplementary Table 3

Supplementary Table 4

Supplementary Table 5

Supplementary Table 6

Supplementary Table 7

Supplementary Table 8
