# Supplementary material for: Comparative analysis of gene family evolution demonstrates expansion of digestive, immunity and olfactory functions in the black soldier fly (Hermetia illucens) lineage
Source: Heredity (Edinb). 2025 Oct 23;135(3):187–98. doi: 10.1038/s41437-025-00805-6 (PMC13031285; doi:10.1038/s41437-025-00805-6)
Supplement: Supplementary file 1 — Supplementary Figure 1-5 [file 41437_2025_805_MOESM1_ESM.pdf]

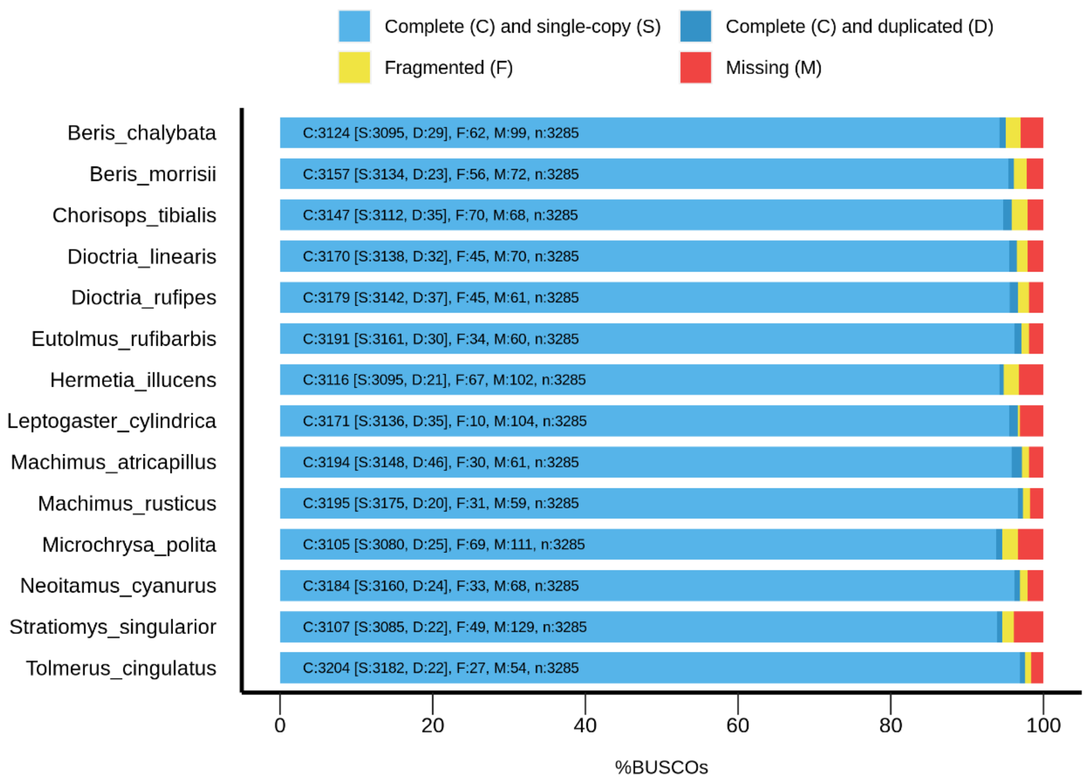

Supplementary Figure 1 BUSCO assessment summary of all reference genomes used in this study. X axis represents the proportion of BUSCO genes compared to all BUSCO genes in the Dipteran database.

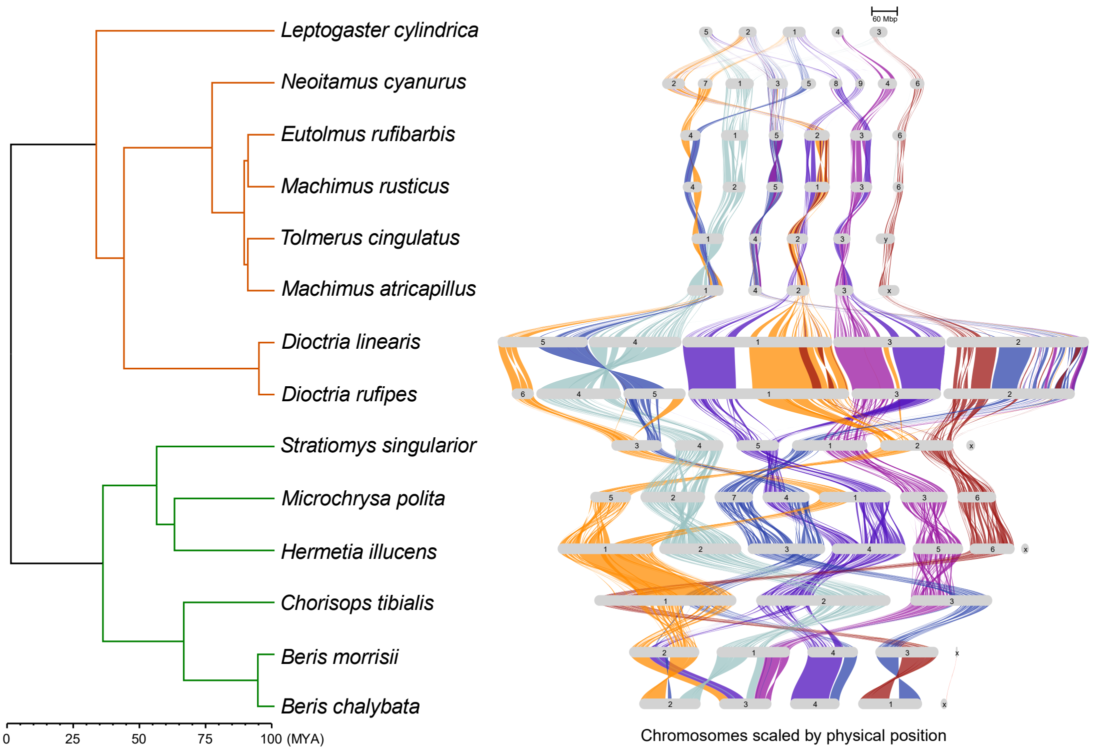

Supplementary Figure 2 Genome-wide synteny scaled by physical length of the chromosomes. Syntenic blocks are aligned based on the order of chromosomes of *Hermetia illucens*. Species tree was rooted using *Drosophila melanogaster* as outgroup which is not shown in the figure. Stratiomyidae and Asilidae families were marked with green and orange branch colours in the phylogeny, respectively. The species tree was calibrated based on estimated divergence time (MYA).

A

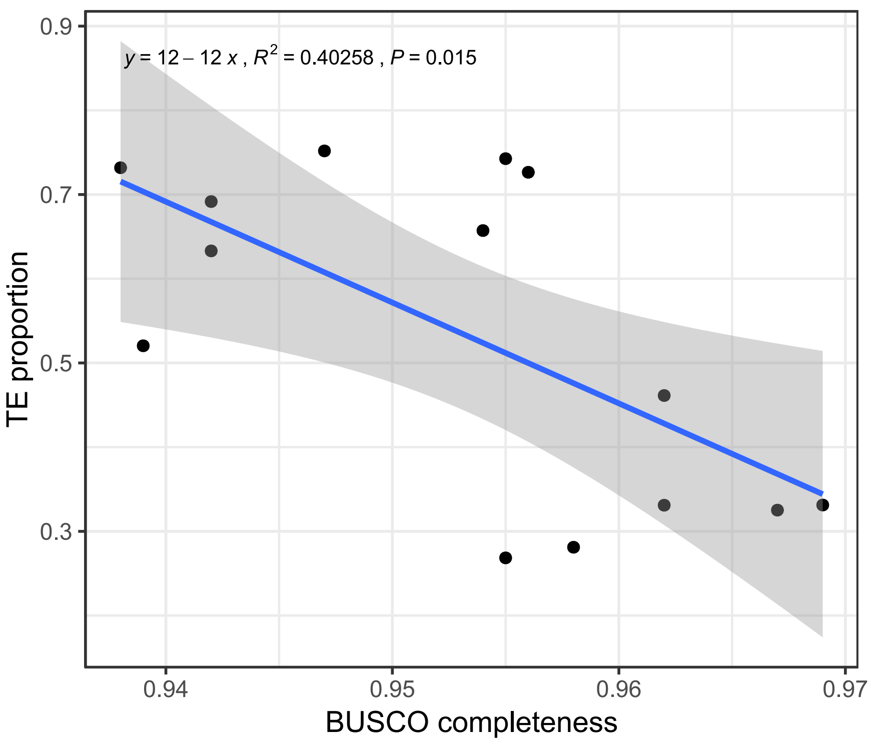

y = 12 − 12 x , R2 = 0.40258 , P = 0.015
TE proportion
0.9
0.7
0.5
0.3
0.94
0.95
0.96
0.97
BUSCO completeness

B

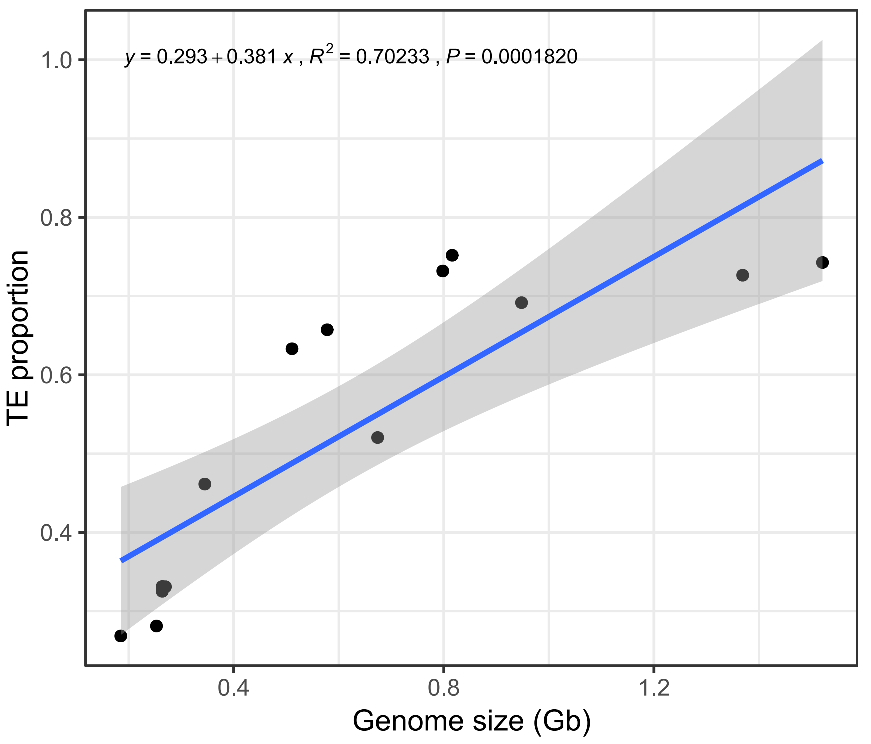

y = 0.293 + 0.381 x , R2 = 0.70233 , P = 0.0001820
TE proportion
1.0
0.8
0.6
0.4
0.4
0.8
1.2
Genome size (Gb)

C

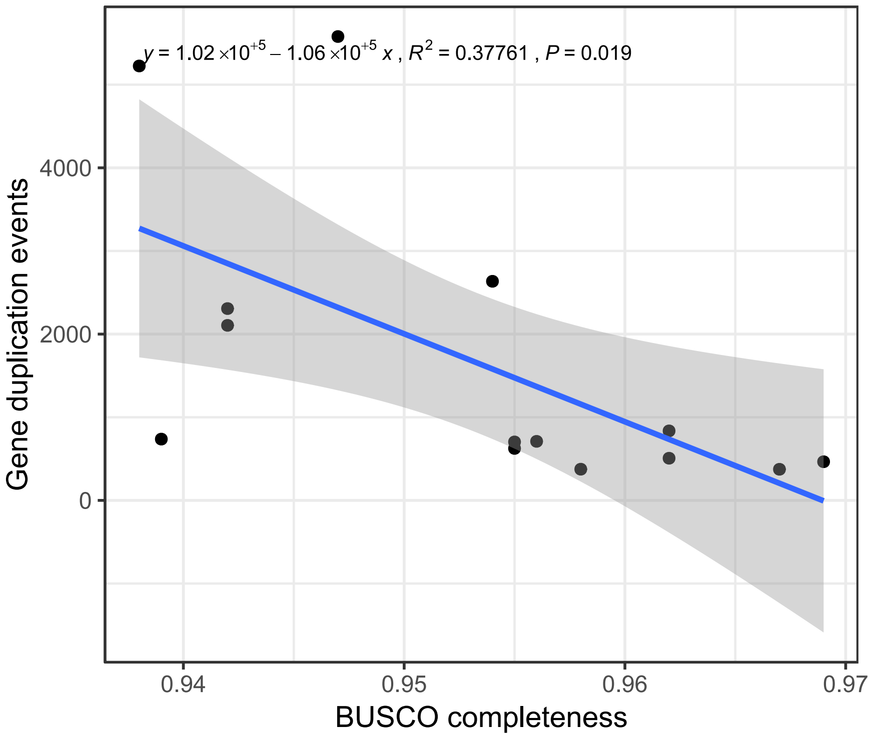

D

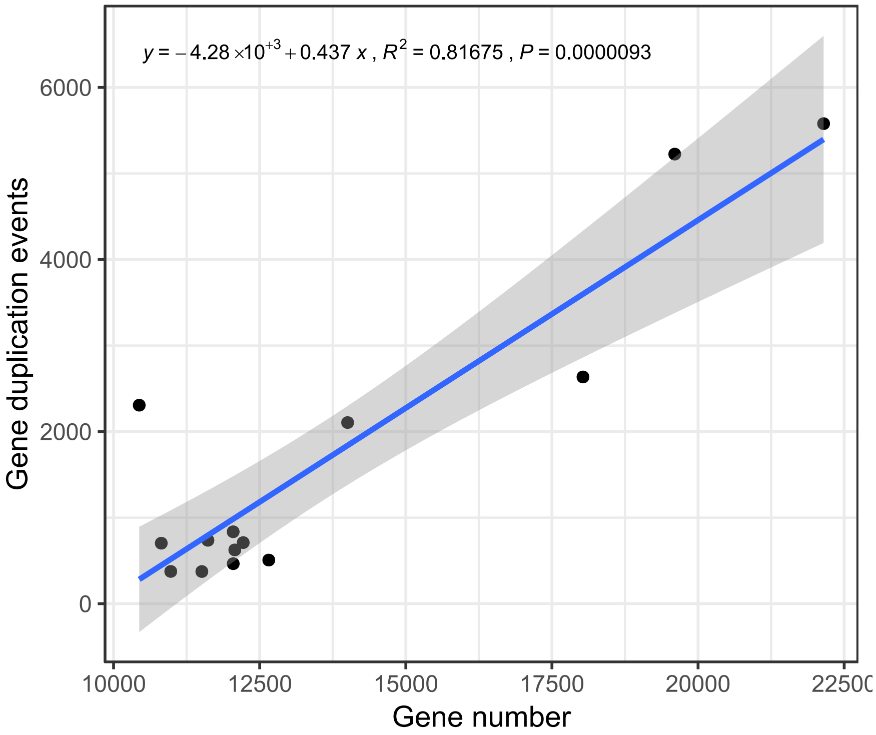

Supplementary Figure 3 Linear regression between: BUSCO completeness level and TE proportion (**A**), genome size and TE proportion (**B**) BUSCO completeness level and gene duplication events (**C**), and gene number and gene duplication

events (**D**). BUSCO completeness levels are represented by the proportion of complete BUSCO genes against all compared BUSCO genes in the Dipteran database (diptera_odb10). Confidence intervals are shown as grey areas around the lines.

A

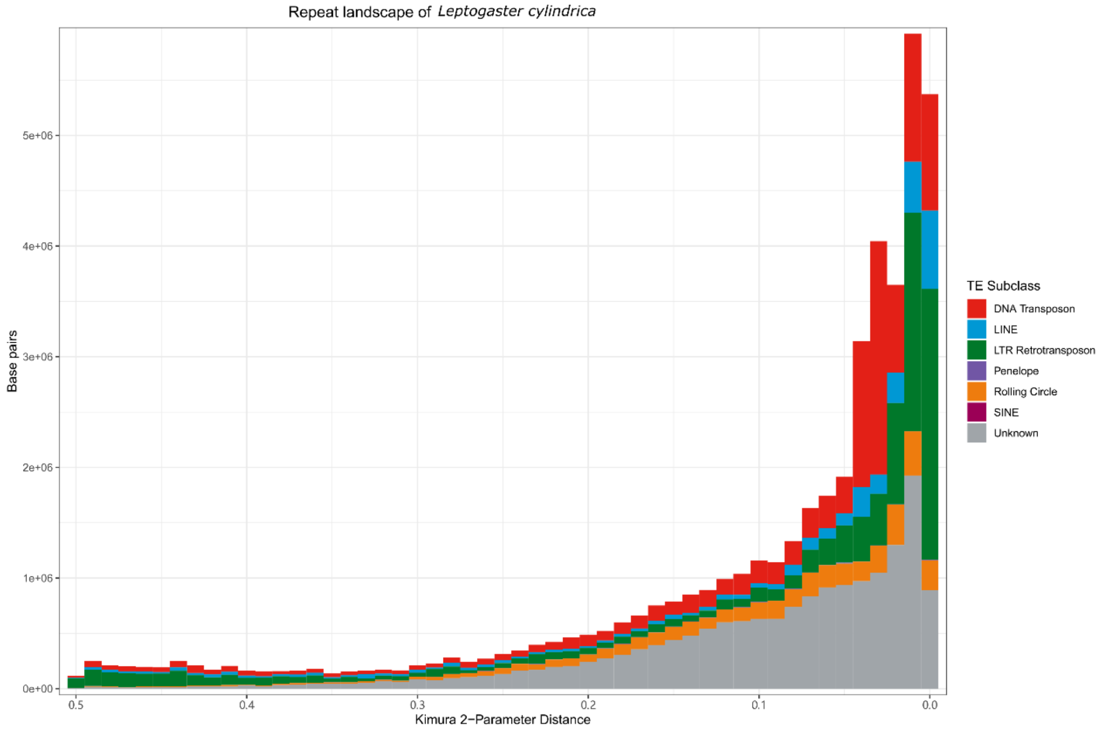
Repeat landscape of Leptogaster cylindrica
Base pairs
5e+06
4e+06
3e+06
2e+06
1e+06
0e+00
0.5
0.4
0.3
0.2
0.1
0.0
Kimura 2−Parameter Distance
TE Subclass
DNA Transposon
LINE
LTR Retrotransposon
Penelope
Rolling Circle
SINE
Unknown

B

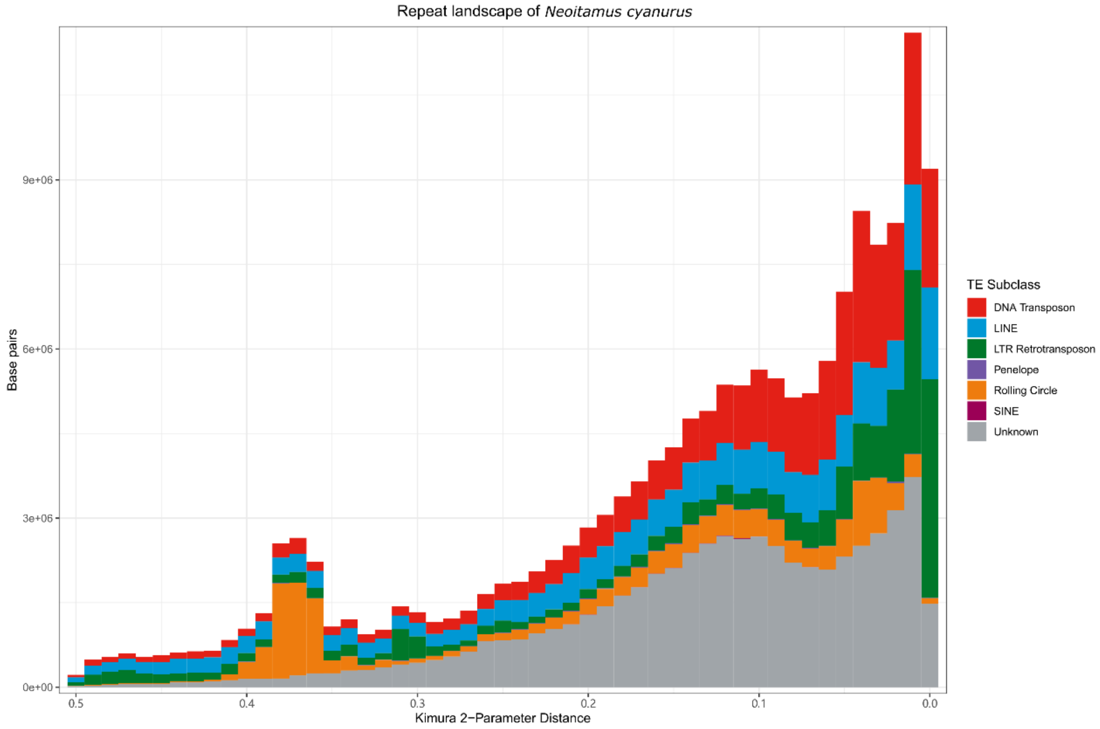
Repeat landscape of Neoitamus cyanurus
Base pairs
9e+06
6e+06
3e+06
0e+00
0.5
0.4
0.3
0.2
0.1
0.0
Kimura 2−Parameter Distance
TE Subclass
DNA Transposon
LINE
LTR Retrotransposon
Penelope
Rolling Circle
SINE
Unknown

C

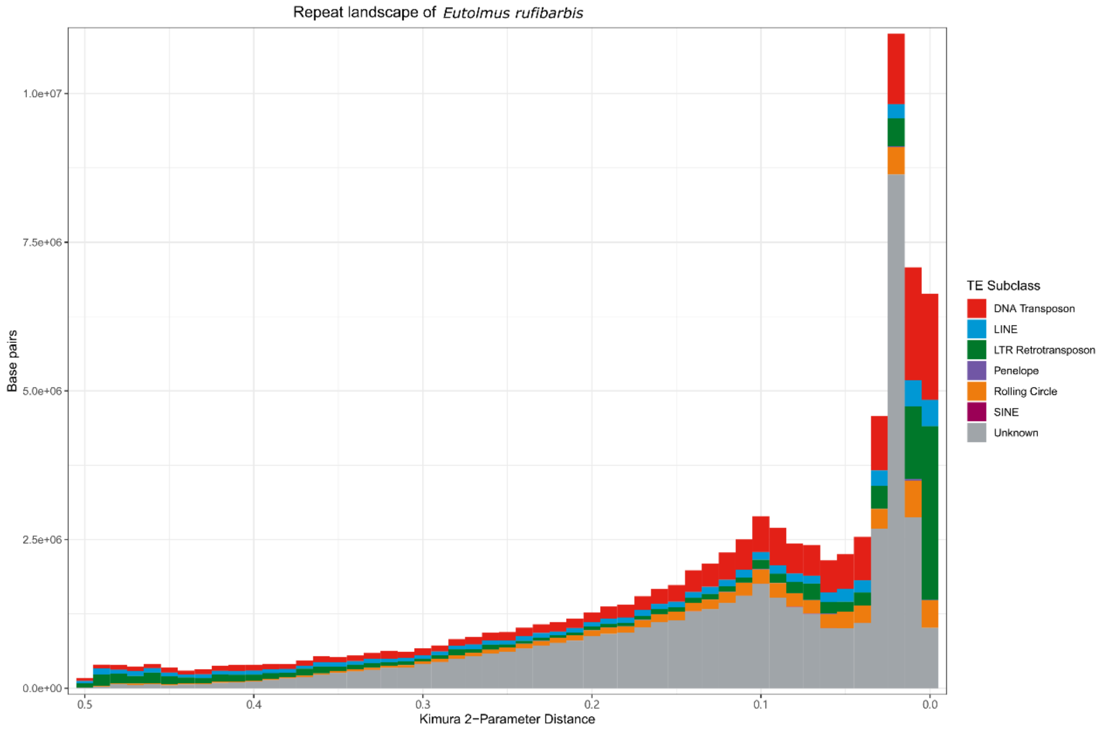

Repeat landscape of *Eutolmus rufibarbis*
Base pairs
0.0e+00
2.5e+06
5.0e+06
7.5e+06
1.0e+07
Kimura 2−Parameter Distance
0.5
0.4
0.3
0.2
0.1
0.0
TE Subclass
DNA Transposon
LINE
LTR Retrotransposon
Penelope
Rolling Circle
SINE
Unknown

D

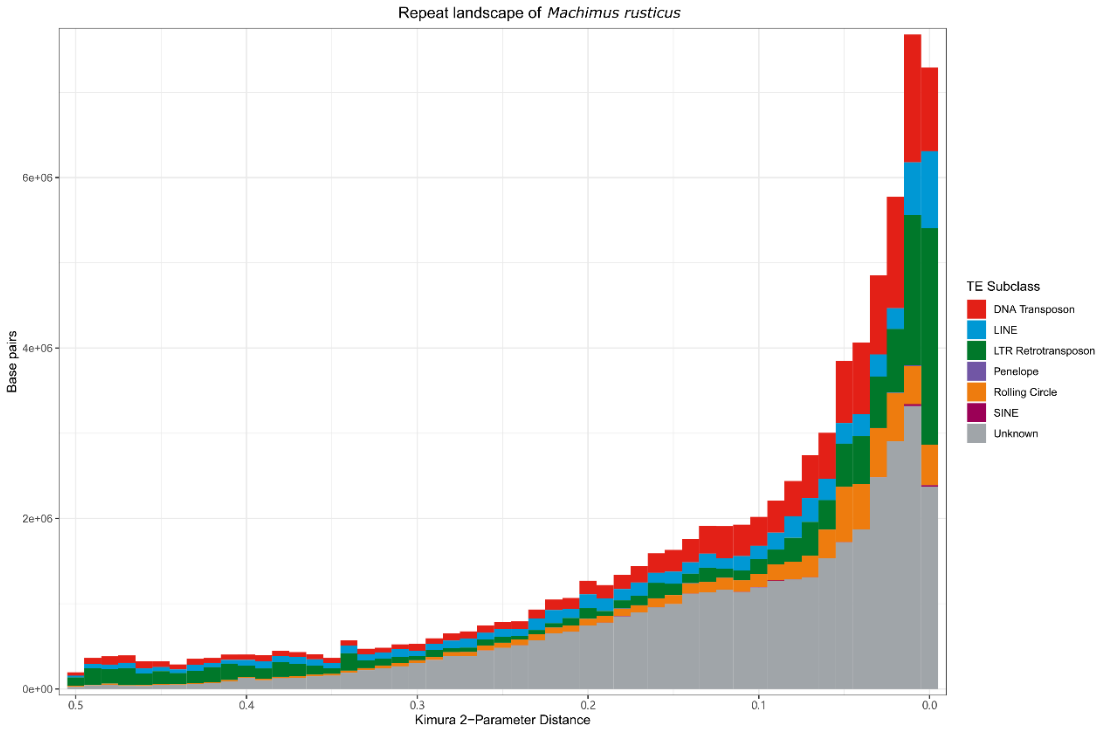

Repeat landscape of *Machimus rusticus*
Base pairs
0e+00
2e+06
4e+06
6e+06
Kimura 2−Parameter Distance
0.5
0.4
0.3
0.2
0.1
0.0
TE Subclass
DNA Transposon
LINE
LTR Retrotransposon
Penelope
Rolling Circle
SINE
Unknown

E

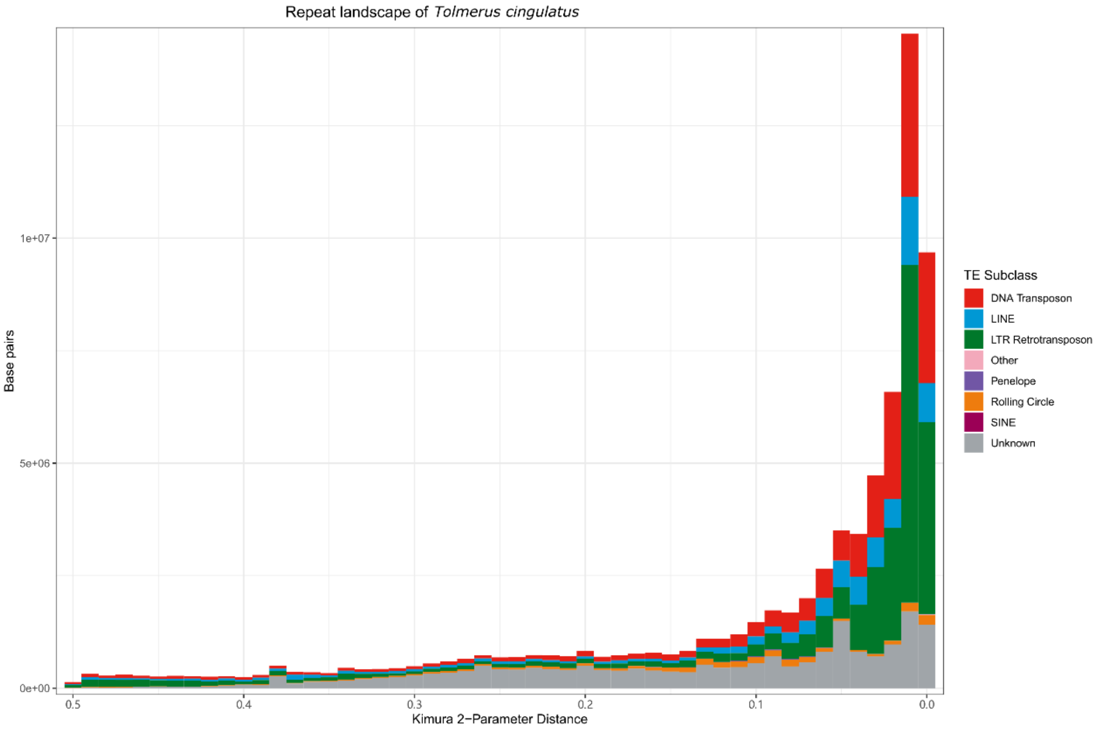
Repeat landscape of *Tolmerus cingulatus*
Base pairs
1e+07
5e+06
0e+00
0.5
0.4
0.3
0.2
0.1
0.0
Kimura 2−Parameter Distance
TE Subclass
DNA Transposon
LINE
LTR Retrotransposon
Other
Penelope
Rolling Circle
SINE
Unknown

F

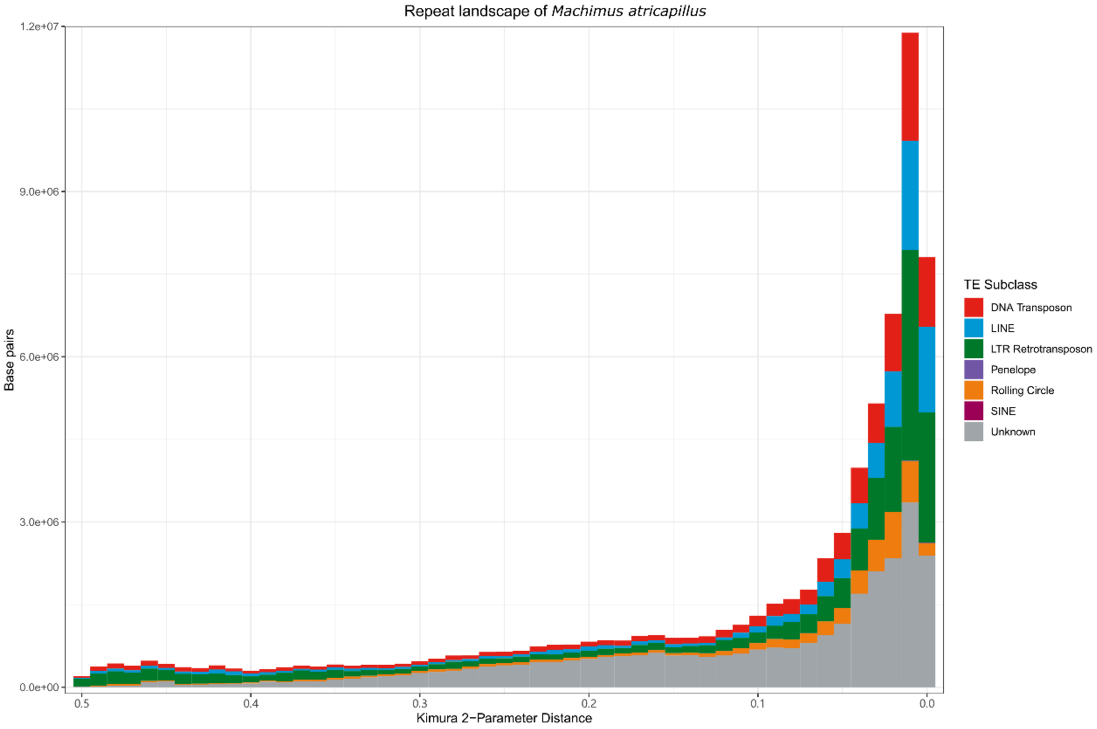
Repeat landscape of *Machimus atricapillus*
Base pairs
1.2e+07
9.0e+06
6.0e+06
3.0e+06
0.0e+00
0.5
0.4
0.3
0.2
0.1
0.0
Kimura 2−Parameter Distance
TE Subclass
DNA Transposon
LINE
LTR Retrotransposon
Penelope
Rolling Circle
SINE
Unknown

G

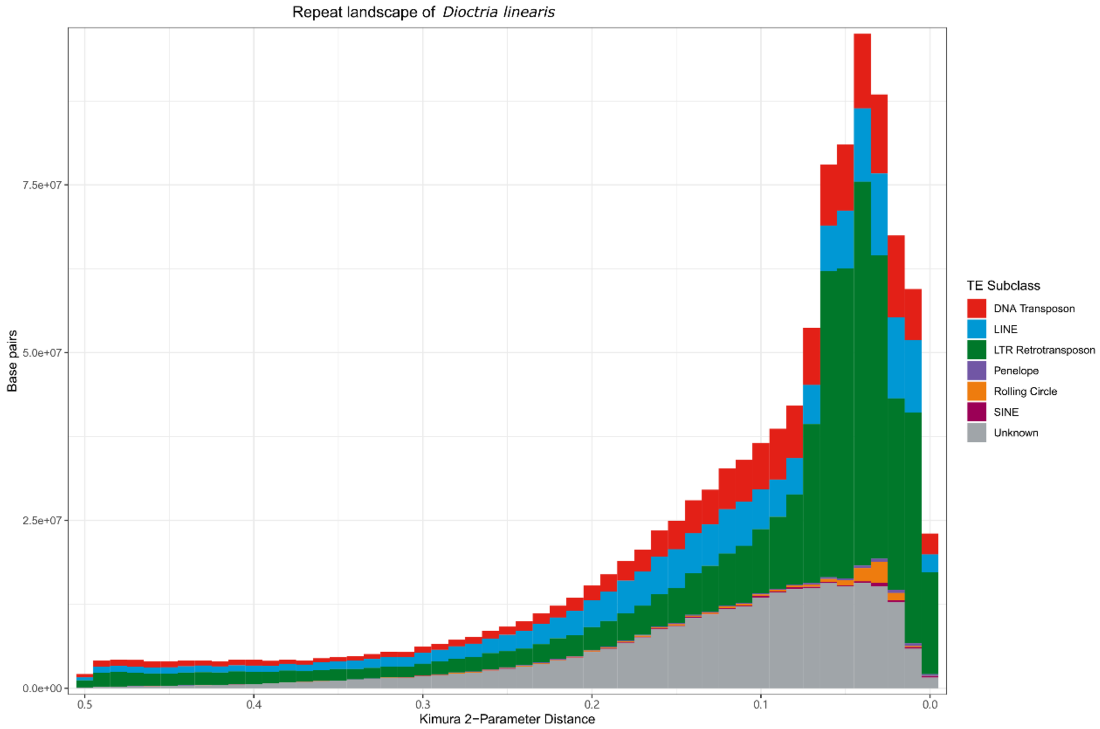

Repeat landscape of *Dioctria linearis*
Base pairs
7.5e+07
5.0e+07
2.5e+07
0.0e+00
0.5
0.4
0.3
0.2
0.1
0.0
Kimura 2−Parameter Distance
TE Subclass
DNA Transposon
LINE
LTR Retrotransposon
Penelope
Rolling Circle
SINE
Unknown

H

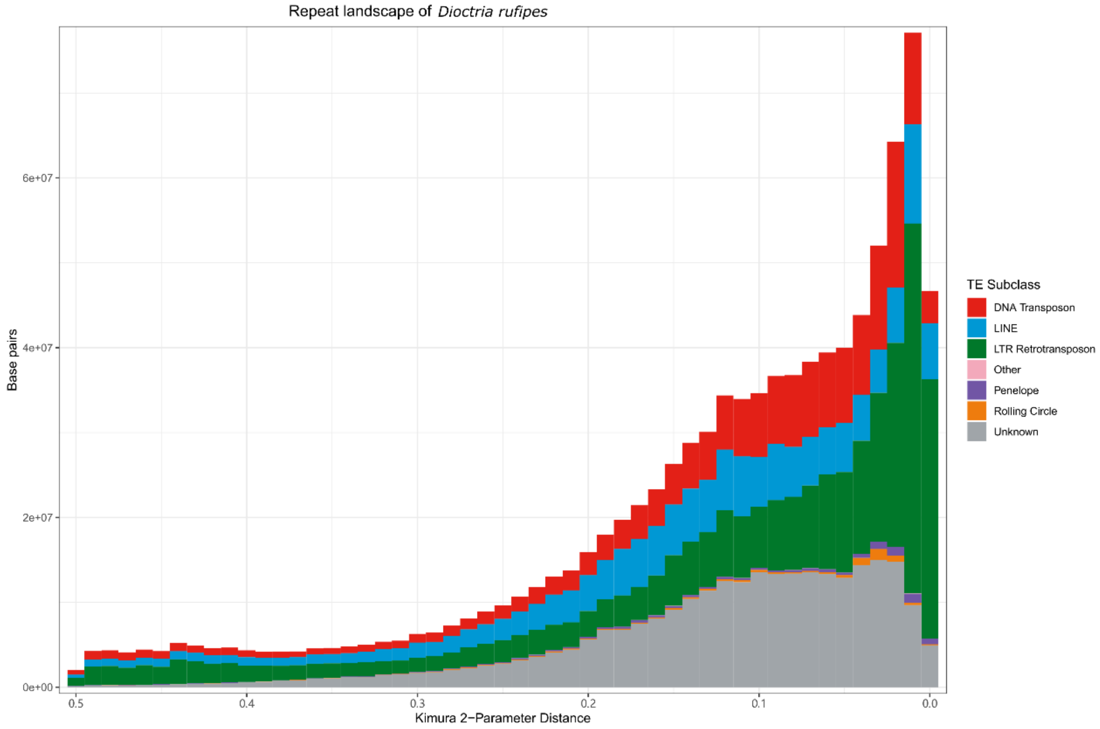

Repeat landscape of *Dioctria rufipes*
Base pairs
6e+07
4e+07
2e+07
0e+00
0.5
0.4
0.3
0.2
0.1
0.0
Kimura 2−Parameter Distance
TE Subclass
DNA Transposon
LINE
LTR Retrotransposon
Other
Penelope
Rolling Circle
Unknown

I

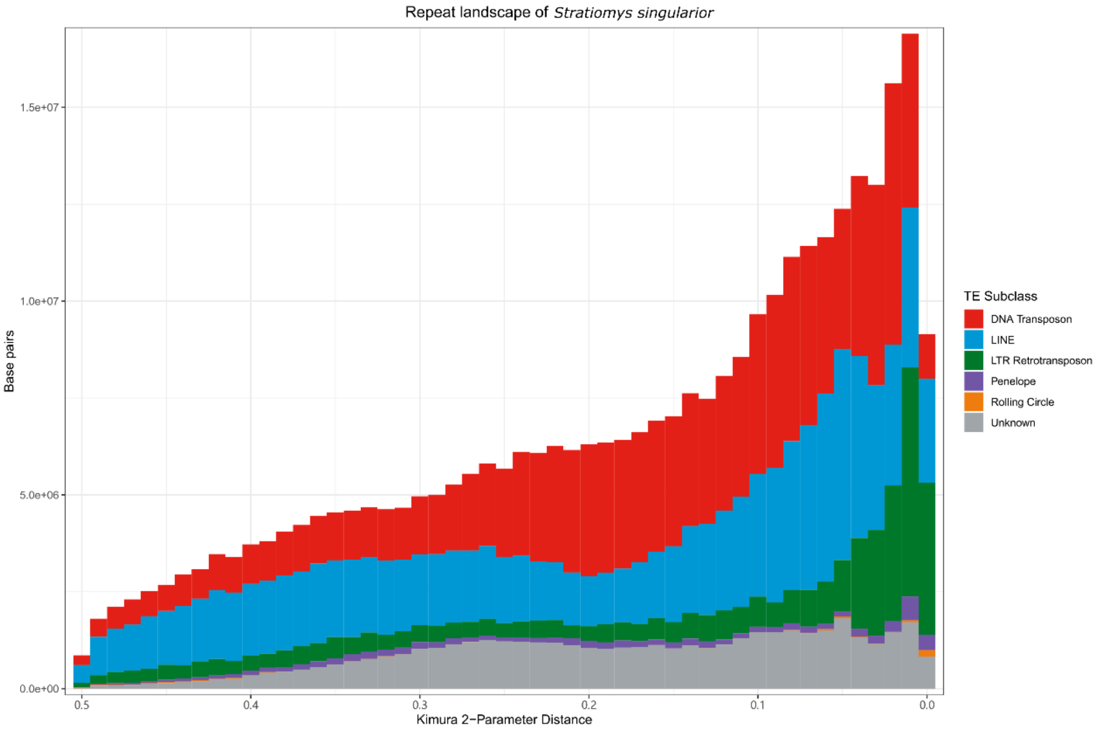
Repeat landscape of *Stratiomys singularior*
Base pairs
1.5e+07
1.0e+07
5.0e+06
0.0e+00
0.5
0.4
0.3
0.2
0.1
0.0
Kimura 2−Parameter Distance
TE Subclass
DNA Transposon
LINE
LTR Retrotransposon
Penelope
Rolling Circle
Unknown

J

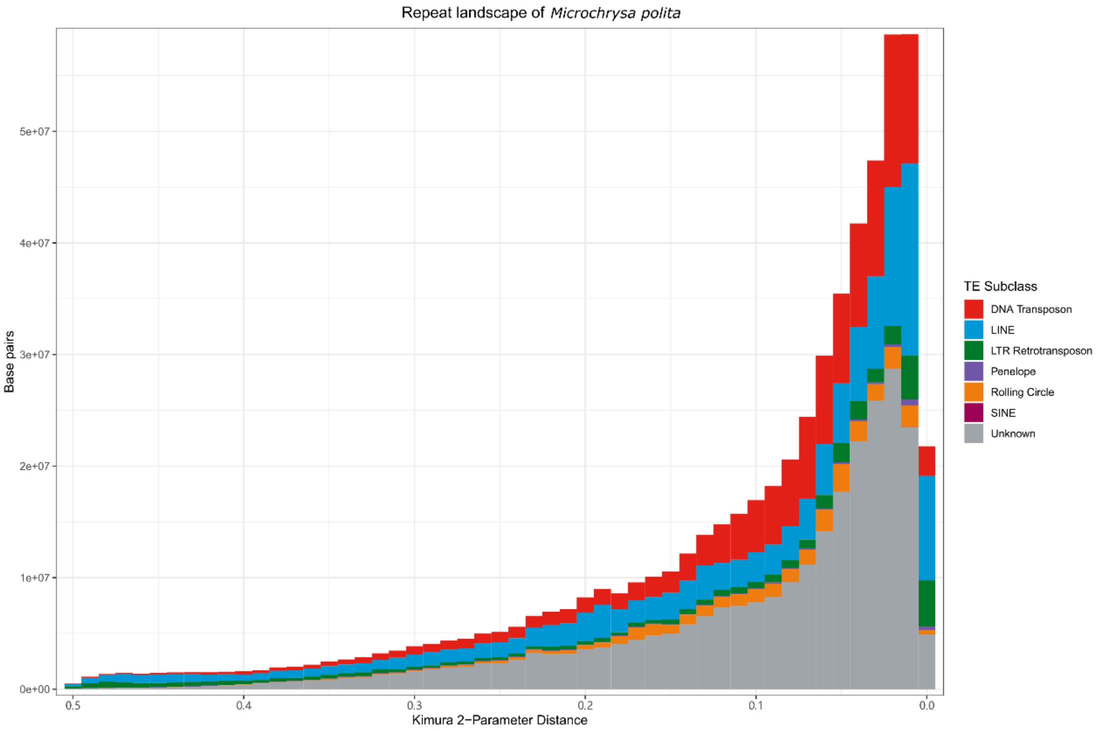
Repeat landscape of *Microchrysa polita*
Base pairs
5e+07
4e+07
3e+07
2e+07
1e+07
0e+00
0.5
0.4
0.3
0.2
0.1
0.0
Kimura 2−Parameter Distance
TE Subclass
DNA Transposon
LINE
LTR Retrotransposon
Penelope
Rolling Circle
SINE
Unknown

K

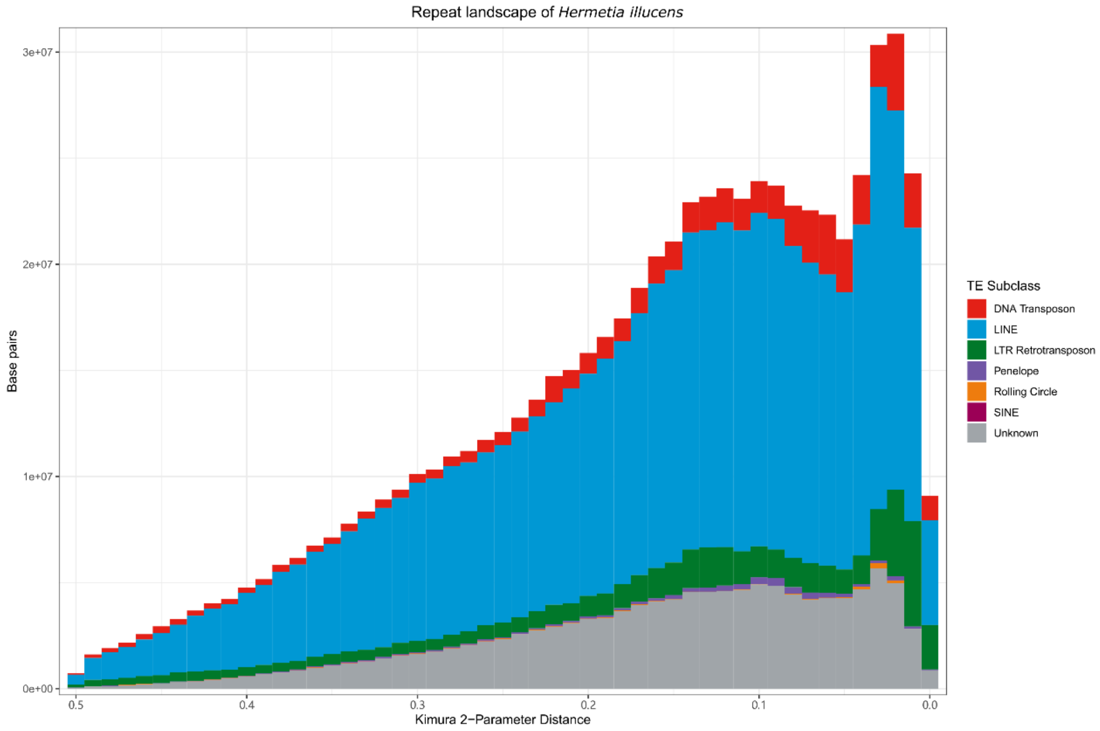
Repeat landscape of *Hermetia illucens*
Base pairs
3e+07
2e+07
1e+07
0e+00
0.5
0.4
0.3
0.2
0.1
0.0
Kimura 2−Parameter Distance
TE Subclass
DNA Transposon
LINE
LTR Retrotransposon
Penelope
Rolling Circle
SINE
Unknown

L

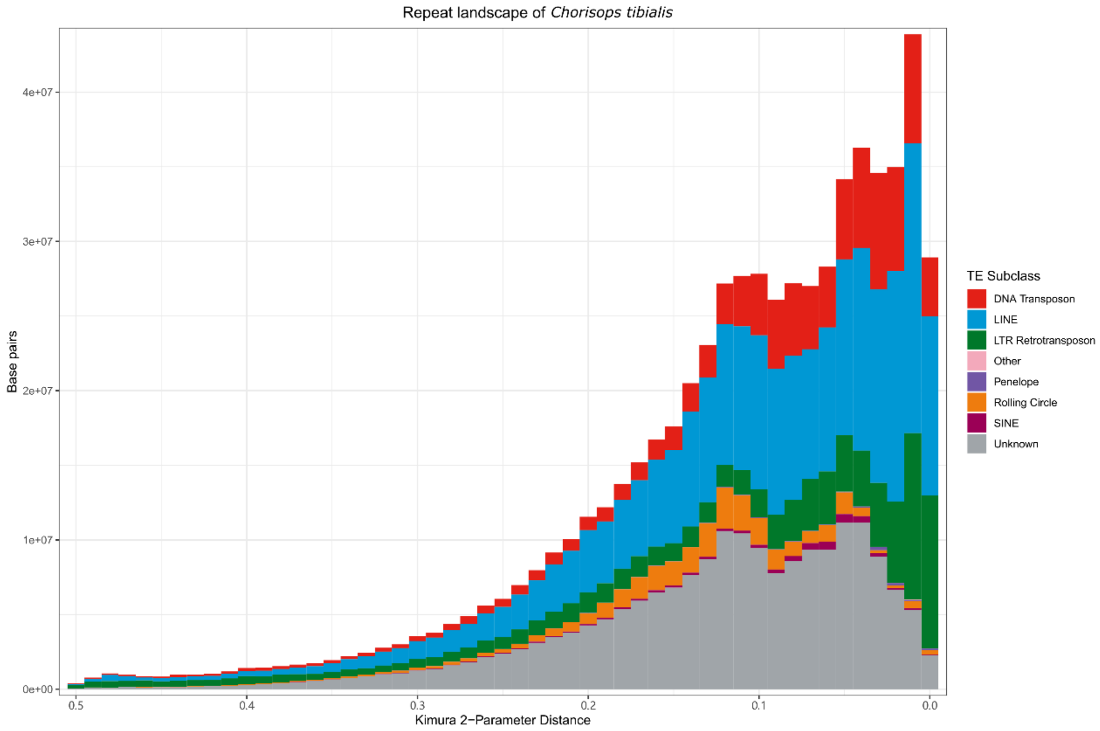
Repeat landscape of *Chorisops tibialis*
Base pairs
4e+07
3e+07
2e+07
1e+07
0e+00
0.5
0.4
0.3
0.2
0.1
0.0
Kimura 2−Parameter Distance
TE Subclass
DNA Transposon
LINE
LTR Retrotransposon
Other
Penelope
Rolling Circle
SINE
Unknown

M

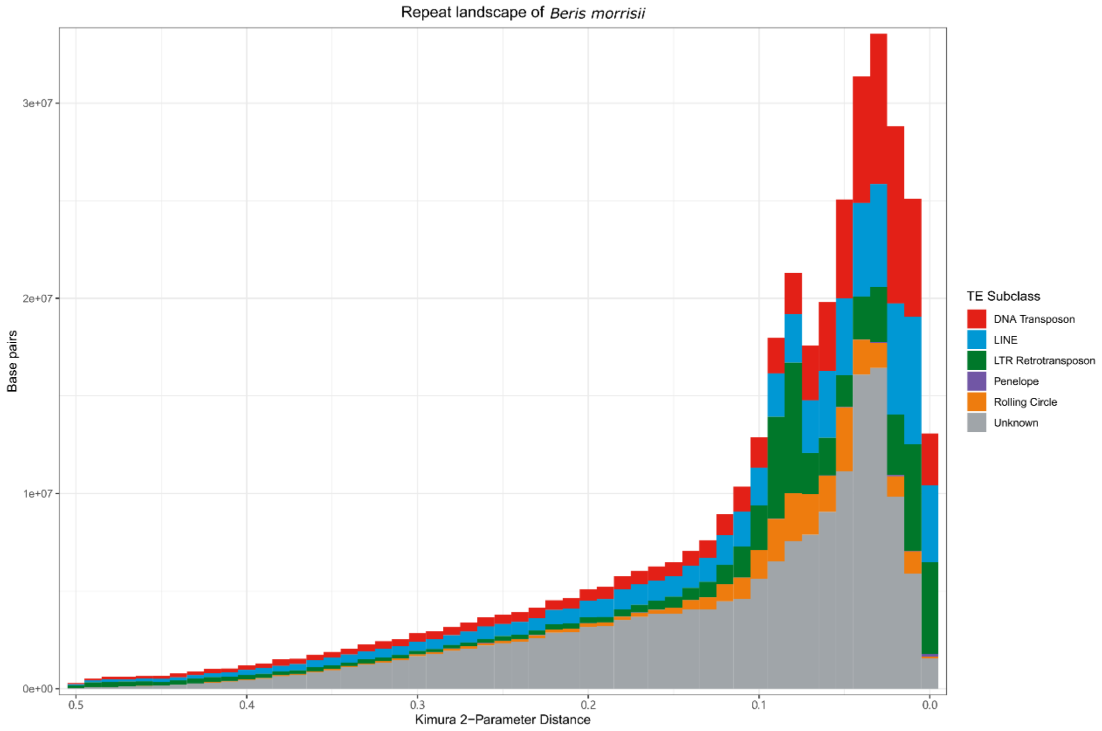

N

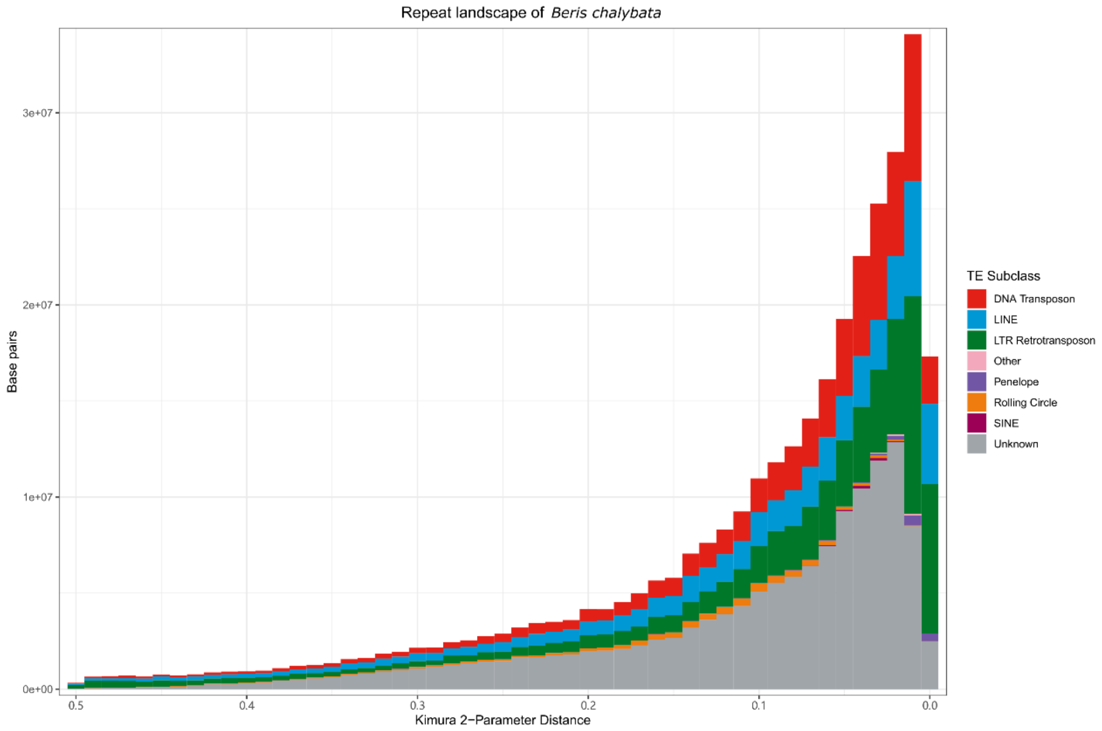

Supplementary Figure 4 Repetitive elements landscape of: *Leptogaster cylindrica* (**A**); *Neoitamus cyanurus* (**B**); *Eutolmus rufibarbis* (**C**); *Machimus rusticus* (**D**); *Tolmerus cingulatus* (**E**); *Machimus atricapillus* (**F**); *Dioctria linearis*

(**G**); *Dioctria rufipes* (**H**); *Stratiomys singularior* (**I**); *Microchrysa polita* (**J**); *Hermetia illucens* (**K**); *Chorisops tibialis* (**L**); *Beris morrisii* (**M**); *Beris chalybata* (**N**). The Kimura 2-Parameter distance (X axis) measures the distance between identified repetitive elements and the consensus sequence. A lower Kimura 2-Parameter distance indicates more recent activity of certain repetitive elements. The abundance of each subclass of repetitive elements is measured by the number of its total base pairs.

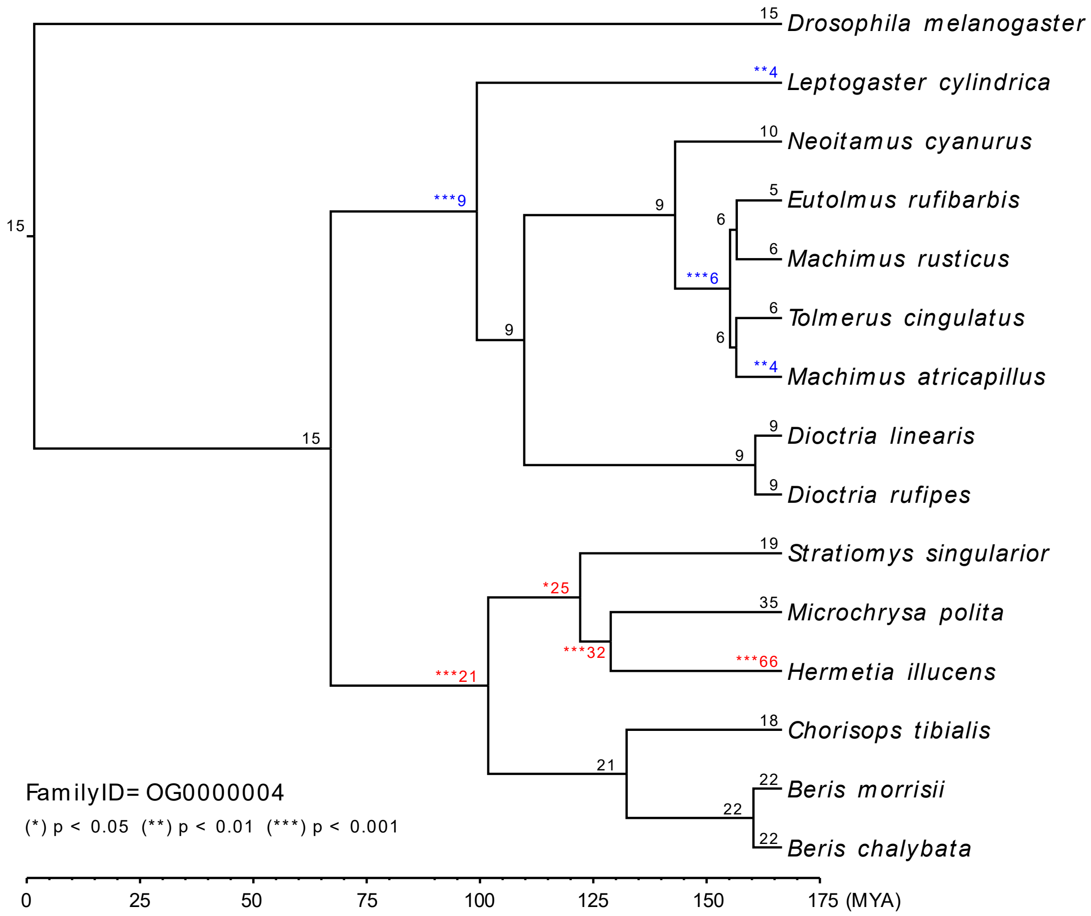

Supplementary Figure 5 The birth-death dynamics of one of the CYP gene families (OG0000004) across the phylogeny. Number of gene copies on each node is marked beside the node. Nodes with significant expansion have red numbers and those with significant contraction have blue numbers. Phylogenetic tree is scaled by estimated divergence time (MYA).
